# Bezafibrate and Cholic Acid-Mediated Lipid Reduction Improves the Cryotolerance of Vitrified In Vivo-Derived Porcine Embryos

**DOI:** 10.3390/ani16172766

**Published:** 2026-09-03

**Authors:** Jiehuan Xu, Mengqian He, Jun Gao, Lingwei Sun, Yuan Zhou, Caifeng Wu, Shushan Zhang, Defu Zhang, Jianjun Dai

**Affiliations:** 1 Shanghai Municipal Key Laboratory of Agri-Genetics and Breeding, Shanghai Engineering Research Center of Breeding Pig, Institute of Animal Science and Veterinary Medicine, Shanghai Academy of Agricultural Sciences, Shanghai 201106, China; jiehuanxu@saas.sh.cn (J.X.); hemengqian@saas.sh.cn (M.H.); gaojun@saas.sh.cn (J.G.); sunlingwei@saas.sh.cn (L.S.); 20260605@saas.sh.cn (Y.Z.); 20150646@saas.sh.cn (C.W.); zhangshushan@saas.sh.cn (S.Z.); zhangdefu@saas.sh.cn (D.Z.); 2 Key Laboratory of Livestock and Poultry Resources Evaluation and Utilization, Ministry of Agriculture and Rural Affairs, Shanghai 201106, China

**Keywords:** in vivo embryo, vitrification, embryo transfer, lipid droplets, bezafibrate, cholic acid, Meishan pig

## Abstract

Background: High lipid accumulation limits the cryotolerance of porcine embryos. This study aimed to identify lipid-lowering combinations during in vivo porcine morulae developing into blastocysts and evaluates their effects on post-warming embryonic development and embryo transfer (ET) pregnancy. Methods: A stable method of in vivo embryo production was established in Meishan pigs to obtain morulae. Morulae were treated with bezafibrate and cholic acid alone, in combination, or with either compound replaced by L-carnitine or niacin, respectively. Treated embryos were vitrified-warmed, followed by determinations of lipid content, cell division and ET. Results: The protocol achieved 95.00% synchronization, with 22.61 embryos obtained per sow in Meishan pigs. The combination of 50 μM of bezafibrate and 50 μM of cholic acid was identified as the optimal non-toxic treatment and produced the lowest lipid droplet content (*p* < 0.05), indicating a synergistic lipid-lowering effect. This treatment improved post-warming embryonic division (*p* < 0.05) and was associated with a numerically higher pregnancy rate after ET (71.43% vs. 50.00%). However, 200 μM of this combination impaired blastocyst formation and delayed post-warming embryonic development. Conclusions: Bezafibrate combined with cholic acid reduced embryonic lipid contents and improved cryotolerance, providing a practical strategy for porcine embryo cryopreservation.

## Simple Summary

Cryopreservation of porcine embryos is essential for germplasm resource preservation and exchanges of livestock breeds. Due to abundant intracellular lipids, porcine embryos are highly susceptible to cryoinjury, which reduces pregnancy rates of vitrified embryo transfer. Developing effective strategies to improve embryo cryotolerance remains a major challenge. This study obtained in vivo morulae from Meishan pigs, an indigenous genetic resource in China, through batch in vivo embryo production (including estrus synchronization, superovulation, artificial insemination, and surgical embryo retrieval). We identified the combination of 50 μM of bezafibrate and 50 μM of cholic acid as the effective treatment that synergistically reduces lipid accumulation in in vivo-derived porcine morulae while maintaining their development to blastocysts. Its enhanced cryo-resistance was manifested by a higher post-warming blastocyst survival rate, hatching rate, and cell proliferation capacity and a 71.43% pregnancy rate after embryo transfer. This practical method is applicable to implementation on farms and provides a technical reference for batch embryo production, cryopreservation and transplantation in pig conservation and breeding programs.

## 1. Introduction

Embryo cryopreservation is a cornerstone of modern livestock breeding and the conservation of valuable animal genetic resources. Embryo vitrification enables the long-term preservation and global distribution of superior pig germplasm resources, while minimizing the cost and risk of animal transportation [1]. In particular, embryo cryopreservation has become an indispensable strategy for establishing livestock gene banks, conserving indigenous breeds, and maintaining genetic diversity [2]. These advantages are particularly important for protecting precious local pig breeds, such as the Meishan pig, whose exceptional reproductive performance [3] and unique genetic characteristics are an important resource for sustainable pig farming and disease-resistant breeding [4]. Despite substantial progress in vitrification techniques, the practical application of porcine embryo cryopreservation remains considerably behind that of cattle and sheep. A series of methodological improvements by the same research group have consistently achieved high post-warming survival rates of porcine embryos [5,6]. However, the routine use of vitrified porcine embryos in commercial breeding programs remains limited [7].

One of the major biological obstacles is the high intracellular lipid content of porcine embryos. Excess lipid droplets (LDs) increase cryo-sensitivity, promoting lipid phase transition and oxidative damage during cooling and warming [8] and ultimately impairing embryo survival, developmental competence, and pregnancy following embryo transfer [9]. To overcome this limitation, invasive mechanical delipation techniques have been developed to physically remove cytoplasmic lipids. However, they inevitably disrupt the zona pellucida and embryonic cytoplasm, increase technical complexity, and raise concerns about embryo biosafety and large-scale practical applications. Consequently, considerable efforts have shifted toward non-invasive metabolic strategies that reduce intracellular lipid accumulation.

Pharmacological regulators, such as L-carnitine and forskolin, which enhance fatty acid β-oxidation or promote intracellular lipolysis, have shown effects in porcine oocytes and IVF embryos [10,11], suggesting that lipolysis before vitrification represents a practical alternative to mechanical delipation [12]. Similarly, pharmacological lipid reduction with L-carnitine or forskolin has been shown to reduce lipid accumulation and improve the cryotolerance of buffalo embryos [13,14]. However, these strategies rely heavily on extended in vitro culturing to achieve sufficient lipid mobilization [15]. While these protocols are effective for in vitro fertilized embryos, their direct application to in vivo-derived embryos raises concerns, as additional in vitro culturing may compromise the superior developmental competence and physiological characteristics of in vivo embryos [16,17]. Therefore, developing a strategy capable of inducing rapid and efficient lipid mobilization within this limited time window represents a critical challenge for improving the cryotolerance of in vivo-derived porcine embryos.

Bezafibrate is a pan-agonist of peroxisome proliferator-activated receptors (PPARs) and a pharmacological activator of lipid oxidative metabolism [18]. By promoting PPAR-dependent transcriptional programs and PGC-1α-associated mitochondrial function, bezafibrate enhances fatty acid oxidation and mitochondrial biogenesis, thereby modulating cellular lipid homeostasis and energy metabolism [19]. In porcine oocytes, bezafibrate-related metabolic regulation has been associated with improved mitochondrial function and reduced oxidative stress [20]. Cholic acid, a primary bile acid synthesized from cholesterol, is another important regulator of lipid and energy homeostasis. Beyond its classical role in lipid emulsification and transport, bile acids also act as metabolic signaling molecules that regulate cellular energy metabolism and mitochondrial function [21]. Bile acid-related pathways have been implicated in the reproductive environment, and cholic acid and its derivatives have been reported to alleviate endoplasmic reticulum stress in embryos [22]. Conversely, altered bile acid signaling has been associated with mitochondrial dysfunction and apoptosis [23]. However, whether cholic acid reduces lipid accumulation in porcine embryos and consequently enhances their cryotolerance remains unknown. Moreover, two candidate alternatives have been provided. L-carnitine enhances mitochondrial fatty acid β-oxidation by facilitating the transport of long-chain fatty acids into mitochondria [24], whereas niacin promotes lipid mobilization through regulating cellular lipid metabolism and redox homeostasis [25].

Given that bezafibrate promotes lipid oxidation through PPAR activation, while cholic acid regulates lipid homeostasis through bile acid signaling, whether this combination has a complementary effect in regulating lipid content remains to be revealed. Their combined application might provide a reasonable pharmacological strategy for mobilizing embryonic lipolysis and improving cryotolerance in cryopreserved embryo transfer.

## 2. Materials and Methods

### 2.1. Reagents

Allyl progesterone, follicle-stimulating hormone (FSH) and human chorionic gonadotropin (hCG), equine chorionic gonadotropin (eCG) and cloprostenol were purchased from Ningbo Sansheng Biotechnology Co., Ltd., Ningbo, China. Zoletil^®^50 was purchased from Virbac, France. Isoflurane was purchased from Shenzhen RWD Life Science Co., Ltd., Shenzhen, China. Nile red was purchased from Shanghai Yuanye Biotechnology Co., Ltd., Shanghai, China. Hoechst 33342 was purchased from Shanghai Beyotime Biotechnology Co., Ltd., Shanghai, China. Other reagents were purchased from Sigma-Aldrich (St. Louis, MO, USA).

### 2.2. Estrus Synchronization, Superovulation and Artificial Insemination

Batch in vivo embryo production includes donor estrus synchronization and superovulation, artificial insemination, and surgical embryo retrieval. Embryo production was conducted in one experimental batch, involving 40 donors. Forty multiparous sows with the last two consecutive records of normal estrus were selected as donors. The donors were fed 20 mg/day of allyl progesterone for 18 d. Each donor was intramuscularly injected with 20 μg of FSH and 100 IU of hCG 42 h after the last allyl progesterone feeding (i.e., day 20) and intramuscularly injected with 200 IU of hCG on day 23. From day 23, a boar was driven twice daily in the sow house to induce estrus. From day 23, artificial insemination was performed with 60 mL of semen per sow on the donors exhibiting the standing reflex, and a second insemination was performed 12 h later.

### 2.3. Surgical Embryo Retrieval

Embryos were obtained surgically 5.5 d after the first artificial insemination. Anesthesia was induced by intramuscular injection of Zoletil^®^50 and maintained by isoflurane inhalation. The donor sow was restrained in a supine position, and its abdomen was disinfected. An incision along the midline of the abdomen was made in the skin at the location of the penultimate and last pairs of nipples, and the subcutaneous tissue, muscles, and peritoneum were separated. A small incision was made at one-third of the way from the uterine body to uterine horn. A sterile latex catheter was inserted and secured with an inflatable balloon on the catheter. A blunt needle was inserted near the fallopian tube at the uterine horn, and 40–50 mL of preheated Tyrode’s lactate–HEPES–polyvinyl alcohol medium (TL-PVA) [26] was injected. The uterine horn was held with the surgeon’s fingers, and the flushing fluid was gently expelled until it all flowed out through the catheter into a 50 mL sterile centrifuge tube. The uterus was sutured and repositioned. The other side of the uterine horn was flushed using the same method. Embryos were retrieved from TL-PVA with a microscope. After surgery, each donor sow was housed in a separate pen and given appropriate antibiotics. The skin wound was disinfected daily with iodine until it healed.

### 2.4. Embryo In Vitro Treatments

Collected in vivo morulae were cultured in vitro for 6 h in North Carolina State University 23 medium (NCSU23) (each 100 mL contained 0.6356 g of sodium chloride, 0.2106 g of sodium bicarbonate, 0.0356 g of potassium chloride, 0.0162 g of potassium dihydrogen phosphate, 0.0294 g of magnesium sulfate heptahydrate, 0.0250 g of calcium chloride dihydrate, 0.1 g of D-glucose, 0.0876 g of taurine, 0.0546 g of hypotaurine, 1 mL of a penicillin–streptomycin solution, and 0.4 g of bovine serum albumin, with a pH value of 7.2) containing 50 μM of bezafibrate, or 50 μM of cholic acid, or their combination, or with either one replaced by 50 μM of L-carnitine or 50 μM of niacin, respectively. Fresh embryos were cultured for 6 h in NCSU23 medium as the control. After 6 h, morulae were morphologically observed under a microscope to determine embryonic development.

### 2.5. Determination of LD Content

Embryos were placed in PBS containing 10 μM of Nile red and incubated at 39 °C for 20 min. After washing three times with PBS, slides were prepared, and the fluorescence of LDs at the largest cross-sectional area of the embryo was observed using a laser scanning confocal microscope. All images were acquired using identical laser power, detector gain, and other acquisition parameters. The embryo area was delineated as the region of interest (ROI) in the Image J software (version 1.53 g, NIH, Bethesda, MD, USA), and the Nile red-positive area within the ROI was quantified using the same thresholding criteria for all groups. An individual embryo was considered the experimental unit for LD staining. For the initial LD-content screening in the embryos, 15 independent experimental replicates were performed (*n* = 15).

### 2.6. Embryo Vitrification and Warming

The methodology referenced previous research [27,28]. Specifically, during each instance of manual operation, 5 embryos were placed in vitrification solution 1 (NCSU23 supplemented with 20% fetal bovine serum, 7.5% dimethyl sulfoxide, and 7.5% ethylene glycol) for 3 min, then they were transferred to vitrification solution 2 (NCSU23 supplemented with 20% fetal bovine serum, 15% dimethyl sulfoxide, 15% ethylene glycol, and 400 mM of sucrose) for approximately 15 s, the vitrified embryos were loaded onto a Cryotop (81111, Kitazato Corporation, Fuji, Japan), and the Cryotop was immediately immersed in liquid nitrogen. The embryos were cryopreserved under liquid nitrogen for 3 months before warming in all experimental replicates. During warming, the embryo loading area of the Cryotop was pulled out of liquid nitrogen and immediately immersed in warming solution 1 (NCSU23 supplemented with 20% fetal bovine serum and 300 mM sucrose) for 5 min, then transferred to warming solution 2 (NCSU23 supplemented with 20% fetal bovine serum and 150 mM of sucrose) and maintained for 5 min, and then transferred to NCSU23 and maintained for 5 min. Warmed embryos were transferred to NCSU23 at 39 °C and 5% CO_2_ for 12 h of recovery.

### 2.7. Determination of Embryo Recovery Rates and Hatching Rates

For embryo recovery and hatching analyses, 90 embryos per group were individually vitrified, warmed, cultured, and evaluated. Each embryo was treated as an individual experimental unit, and the outcomes were recorded as binary variables. Each embryo was observed morphologically with a microscopy at post-warming 6 h and 12 h, as a binary outcome (recovered or not recovered). For vitrified-warmed early blastospheres, those recovered from a shrunken vitrified state with relatively dense cytoplasm and without loose or empty cell clusters were considered viable. For vitrified-warmed blastocysts, if the cytoplasm recovered from a shrunken and vitrified state and a cavity of the blastocyst appeared, the blastocyst was considered viable. The 6 h recovery rate and 12 h hatching blastocyst rate were statistically analyzed.

### 2.8. Determination of Blastocyst Cell Numbers

Embryos were fixed with 4% paraformaldehyde for 1 h and permeabilized with 0.1% Triton-X 100 for 10 min, then incubated with Hoechst 33342 for 15 min, washed three times with PBS, prepared as slides, and observed under a laser scanning confocal microscope. The number of cell nuclei was counted by the Image J software to reflect the number of blastocyst cells. Individual embryos were considered the experimental units for the LD area–nuclei number analysis, with 30 independent experimental replicates performed (*n* = 30).

### 2.9. Surgical Embryo Transfer

ET recipients were orally administered 20 mg of allyl progesterone daily from days 1 to 18. On day 19, 0.2 mg of cloprostenol was administered intramuscularly, followed by intramuscular administration of 1000 IU of eCG on day 20 and 500 IU of hCG on day 23. Ovulation was expected to occur within 1–2 days after hCG injection.

After 12 h of in vitro recovery, vitrified-warmed embryos were transferred into the uteri of 6.25-day-synchronized estrus sows. Anesthesia and the surgical procedure referred to embryo retrieval. Briefly, a small incision was made with a fine needle at the uterine horn, about one-third of the way from the ovary. The ET catheter was inserted 3–4 cm into the uterus through the incision to transfer 1 mL of NCSU23 containing embryos. It was confirmed that there was no bleeding point, and the uterine was reinserted into the abdominal cavity. The peritoneum, muscle, fat, and skin were sutured separately. Each recipient was kept in a single pen with proper postoperative care. Ultrasonographic examination of the uterus 30 d later confirmed pregnancy if gestational sacs were observed.

### 2.10. Statistical Analysis

Statistical analyses were performed using SPSS Statistics (version 22, IBM, USA). Quantitative data are expressed as the mean ± standard deviation (SD). The normality of quantitative data was assessed using the Shapiro–Wilk test before parametric analyses. All datasets used for parametric tests met the assumption of normality (*p* > 0.05). For comparisons between two independent groups, Student’s *t*-test was performed. For comparisons among three groups, one-way analysis of variance (ANOVA) followed by Tukey’s multiple-comparison test was used. Binary developmental outcomes (re-expansion and hatching rates) were analyzed using Pearson’s chi-square (χ^2^) test, whereas pregnancy rates were analyzed using Fisher’s exact test. Differences were considered statistically significant at *p* < 0.05.

## 3. Results

### 3.1. The Efficiency of Batch In Vivo Embryo Production in Meishan Pigs

The results of batch embryo production are listed in Table 1. A synchronization rate of 95.00%, a fertility rate of 100.00% and an average of 22.61 embryos per sow were gained through batch embryo production in Meishan pigs. Under morphological observation, 90.92% were morulae, and the remainder were embryos from potentially fertilized to 8-cell stages. These in vivo non-morula embryos were incubated in NCSU23 for 12 h. All 8-cell stage embryos were capable of developing into blastocysts, while no further development was observed in the potentially fertilized, 2-cell stage, or 4-cell stage embryos.

### 3.2. Bezafibrate and Cholic Acid Exhibited a Synergistic Effect in the Lipid Reduction in Porcine Embryos

**Figure 1:**
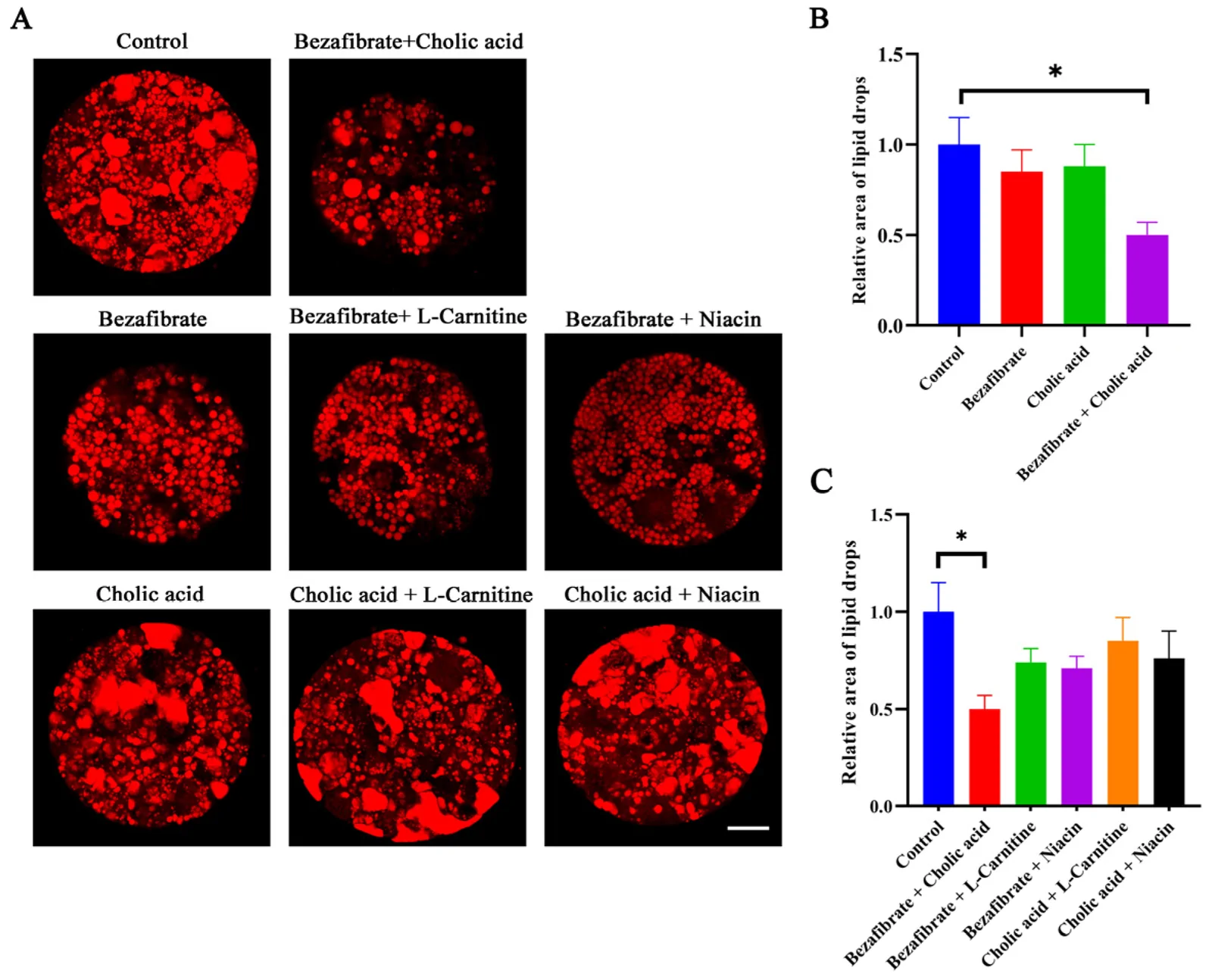
Determination of lipid content in embryos. (**A**) Fluorescence of LDs in embryos stained by Nile red. Embryos were treated with 50 μM of bezafibrate, or 50 μM of cholic acid, or their combination, or either one replaced by 50 μM of L-carnitine or 50 μM of niacin, respectively; bar = 20 μm. (**B**,**C**) Statistical analysis of (**A**) (*n* = 15). *: *p* < 0.05.

Whether in the control, treated with bezafibrate and cholic acid alone, or treated with them in combination, 100% of morulae developed into blastocysts or early blastocysts within 6 h. Significant variation in lipid content was not observed following treatment with bezafibrate or cholic acid alone. In contrast, when the two were incubated together, the LD content decreased significantly (*p* = 0.018) (Figure 1A,B), confirming a synergistic effect between bezafibrate and cholic acid. To verify whether this synergistic effect was specific to the bezafibrate–cholic acid combination, one compound was replaced with L-carnitine or nicotinic acid, respectively. The results confirm that replacing either bezafibrate or cholic acid with L-carnitine or niacin abolished this synergistic effect, as their reductions in embryonic LD content were not significant (*p* > 0.05) (Figure 1A,C).

### 3.3. Excessive Bezafibrate Combined with Cholic Acid Treatment Hindered Early Embryo Development

**Figure 2:**
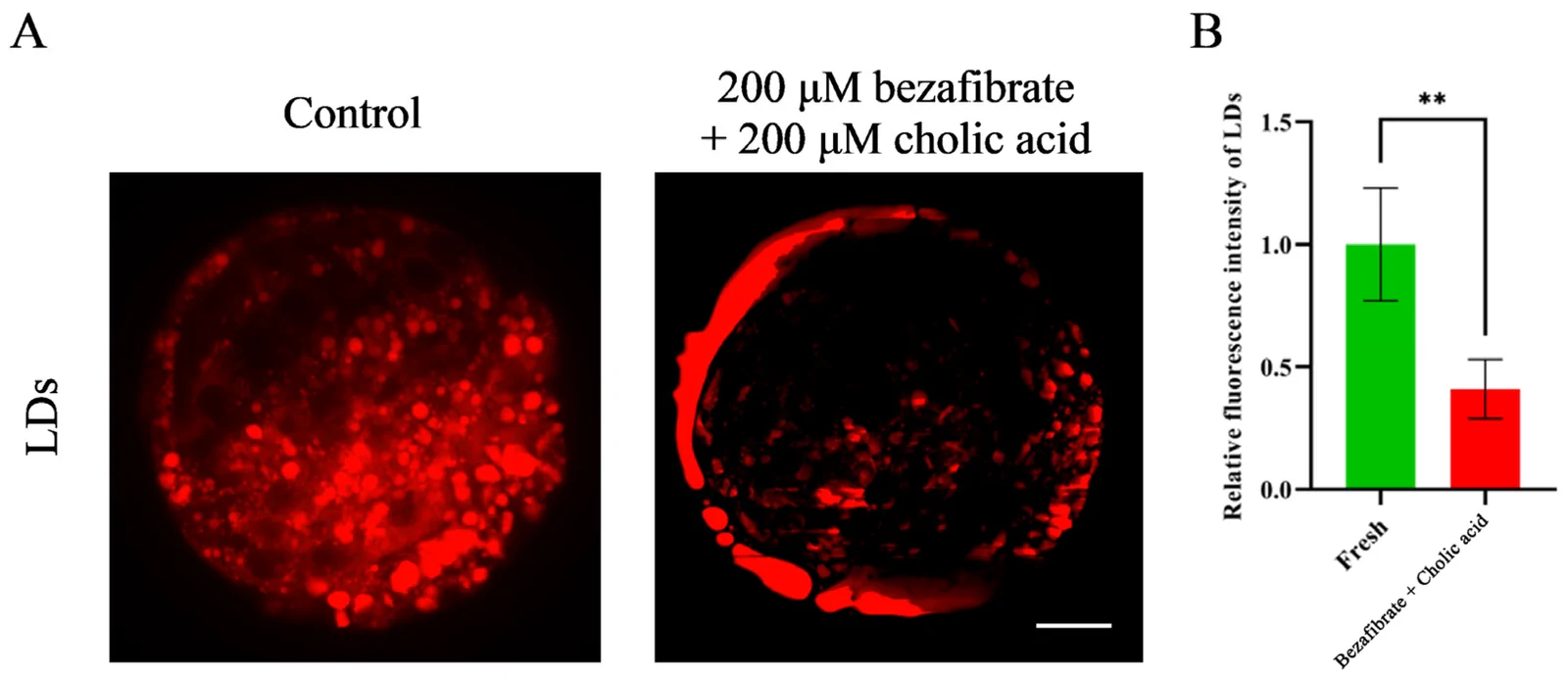
Effect of excessively high concentrations of bezafibrate combined with cholic acid on lipid clearance in embryos. (**A**) LD content in embryos stained by Nile red; bar = 20 μm. (**B**) Statistical analysis of relative LD areas (n = 15). **: *p* < 0.01.

Morulae were treated with 200 μM of bezafibrate combined with 200 μM of cholic acid for 6 h to further examine how far the lipid clearance could reach and its influence on embryonic development. As a result, the cytoplasm of early blastocysts became lighter in color under morphological observation. However, compared to this combination in 50 μM concentration, 200 μM of bezafibrate combined with 200 μM of cholic acid reduced the proportion of morula embryos that developed into blastocysts (57/90) (63.33% vs. 100.00%). Meanwhile, after this 200 μM combined treatment, the LD content was significantly lower than that of the control group (*p* < 0.01), indicating a further enhanced lipid clearance capacity (Figure 2). A total of 6 h after vitrification-warming, the re-expansion rate of blastocysts treated with 200 μM of bezafibrate combined with 200 μM of cholic acid reached 78.57% (33/42), while the hatching rate after 12 h was 33.33% (14/42).

### 3.4. Lipid Pre-Reduction Enhanced Recovery and Hatching of Vitrified-Warmed Porcine Embryos

**Figure 3:**
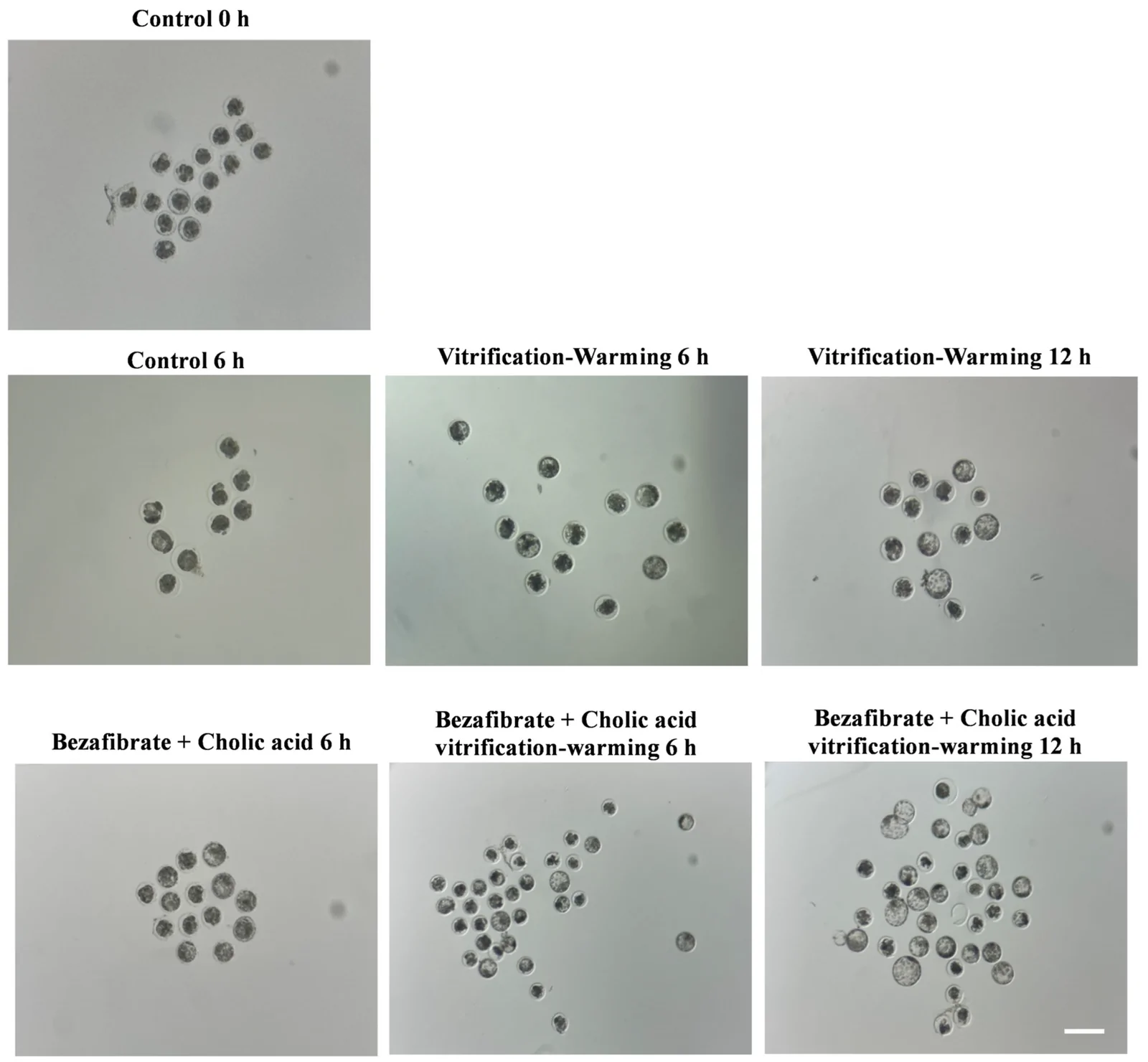
Recovery and hatching of vitrified-warmed blastocysts. Bar = 200 μm.

As determined by morphological observation 6 h after warming, blastocysts pretreated with 50 μM of bezafibrate and 50 μM of cholic acid before vitrification exhibited a higher re-expansion rate than that of the vitrified-warmed control (77.78% [70/90] vs. 58.89% [53/90], χ^2^ = 7.42, *p* = 0.006) (Figure 3). At 12 h after warming, the hatching rate of bezafibrate combined with cholic-acid-pretreated embryos was also higher than that of the vitrified-warmed control embryos (66.67% [60/90] vs. 42.22% [38/90], χ^2^ = 10.11, *p* = 0.001) (Figure 3).

### 3.5. Lipid Pre-Reduction Enhanced Post-Warming Embryonic Cell Numbers

**Figure 4:**
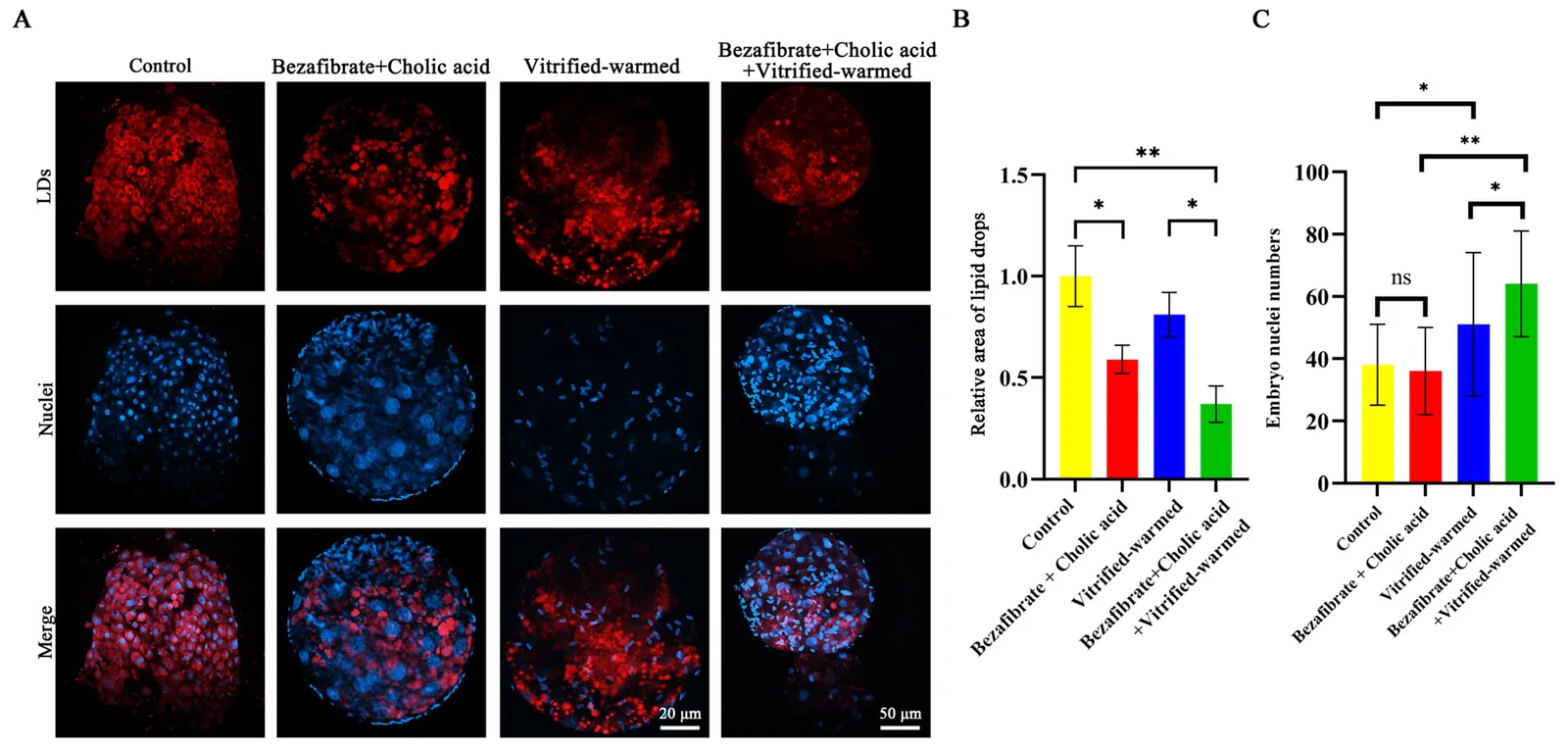
Determination of lipid content and cell division of embryos before and after cryopreservation. (**A**) Fluorescence of LDs and cell nuclei in embryos stained by Nile red and Hoechst 33342. (**B**) Statistical analysis of relative LD contents (n = 30). (**C**) Statistical analysis of cell nuclei numbers (n = 30). *: *p* < 0.05. **: *p* < 0.01. “ns”: *p* ≥ 0.05.

Colocalization analysis of lipid droplets and cell nuclei showed that compared with the control embryos, embryos treated with 50 μM of bezafibrate combined with 50 μM of cholic acid exhibited a significant reduction in lipid droplet content (*p* < 0.05), whereas the number of cell nuclei remained largely unchanged (*p* > 0.05) (Figure 4). Following vitrification and warming, the control embryos exhibited a significant increase in cell nuclei number compared with their pre-vitrification counterparts (*p* < 0.05), indicating continued cell division during post-warming recovery.

Notably, vitrified-warmed embryos pretreated with bezafibrate combined with cholic acid retained significantly lower LD contents than vitrified-warmed embryos. At the same time, the number of cell nuclei was significantly higher in bezafibrate combined with cholic-acid-pretreated embryos than in the control vitrified-warmed embryos (*p* < 0.05) and markedly higher than their pre-vitrification levels (*p* < 0.01). These findings collectively demonstrate that embryos pretreated with bezafibrate combined with cholic acid exhibited faster cell division after cryopreservation.

### 3.6. Bezafibrate Combined with Cholic Acid Was Associated with a Numerically Higher Pregnancy Rate Following Vitrified-Warmed ET

ET was performed 12 h after embryo warming, with 30 embryos transferred per recipient. In total, 120 vitrified-warmed embryos were transferred to four recipients, whereas 210 bezafibrate and cholic-acid-pretreated vitrified-warmed embryos were transferred to seven recipients. Pregnancy was confirmed by ultrasonography 30 d later. The pregnancy rate of recipients receiving embryos pretreated with bezafibrate and cholic acid before vitrification was 71.43% (5/7), which was numerically higher than that of control recipients receiving vitrified-warmed embryos (50.00% [2/4]). However, the difference was not statistically significant (two-sided Fisher’s exact test, *p* = 0.57).

## 4. Discussion

In the present study, we have demonstrated that short-term lipid preconditioning of in vivo-derived porcine morulae with bezafibrate and cholic acid before vitrification improved subsequent embryonic competence and cryotolerance. Furthermore, transient pharmacological modulation of lipid contents can be implemented without interfering with embryonic development at the level of embryonic morphology. Following vitrification and warming, preconditioned embryos exhibited improved developmental recovery and a more active embryonic cell division, suggesting that an appropriate reduction in excessive lipid accumulation before vitrification preserved cellular integrity and developmental capacity against cryo-stress. A numerically higher pregnancy rate was observed after ET following this pretreatment, although this difference did not reach statistical significance. Together, these findings demonstrate that short-term bezafibrate and cholic acid treatment was a feasible approach for improving the cryotolerance of porcine embryos.

Developing a non-invasive method using the physiological lipid-lowering mechanism of embryos represented a feasible strategy to improve embryo cryoprotection [29]. In early studies, intracellular lipids in porcine embryos or oocytes were removed by centrifuging and polarizing lipids, combined with invasive microscopic liposuction, to improve cryoprotection [30]. However, embryos are sensitive to the in vitro environment, and this method inevitably damages the zona pellucida and cytoplasm, leading to cytoskeleton damage and the risk of pathogenic microorganism infection [31]. Furthermore, it is highly dependent on the microsurgical system and operator experience, making it difficult to meet the needs of breeding and production in farms [32]. Finding suitable application strategies for farm environments is of practical significance for introducing cryopreserved embryos into pig production. During vitrification and warming, rapid changes in temperature, osmotic pressure, and membrane phases exert enormous stress on the cell membrane and intracellular organelles [33]. Since lipid droplets and membrane lipids are closely related to cellular energy storage and membrane homeostasis, excessive lipid accumulation may increase the vulnerability of embryos to these stresses [34]. A previous study suggested that a moderate reduction in excessive lipid stores before vitrification established a cellular state to withstand the metabolism and membrane perturbations associated with cryopreservation [35].

Notably, based on our previous practice with Meishan pigs, in vivo-derived morulae rapidly develop into blastocysts within 4–6 h in NCSU23 or porcine zygote medium-3 (PZM-3), of which embryonic development was substantially faster than that of IVF embryos [36]. At the same time, current embryo vitrification protocols generally favor unexpanded blastocysts over expanded or hatching blastocysts [37], as an expansion of the cavity of the blastocyst increases the volume of water that needs to be rapidly removed during cryopreservation. Also, an intact zona pellucida provides better structural protection influencing embryo integrity during cryopreservation [38]. This limits the endpoint of in vitro maintenance of embryos [39]. Consequently, this narrow developmental window leaves only a few hours for metabolic intervention before embryos reach the optimal stage for vitrification.

The choice of cholic acid and bezafibrate as a combined lipid regulation intervention is based on their complementary metabolic properties. Cholic acid is involved in lipid dissolution and transport and regulates cellular lipid homeostasis. In contrast, bezafibrate, as a PPAR agonist, regulates multiple aspects of lipid metabolism, including fatty acid utilization and lipid turnover. Therefore, these two compounds might affect embryonic lipid homeostasis through partially different but potentially complementary mechanisms. The expression of key genes or proteins involved in related signaling pathways, however, was not evaluated by Realtime PCR or Western blotting in this study.

One possible explanation for the observed effect might involve interactions between PPAR signaling and bile acid-responsive pathways mediated by farnesoid X receptor (FXR) and G protein-coupled bile acid receptor 1 (TGR5/GPBAR1) [40]. PPAR coactivator-1 (PGC-1) might represent a potential regulatory node linking these pathways to mitochondrial function and lipid utilization [41]. In contrast, L-carnitine primarily supported mitochondrial fatty acid utilization, whereas niacin regulated lipid metabolism through distinct mechanisms [24,42]. These differences might help explain why the combination of bezafibrate and cholic acid showed a greater lipid-reducing effect than those involving L-carnitine or niacin [43]. However, signaling pathway was not directly examined in the present study and therefore remains a potential mechanism that warrants further investigation.

The combined treatment significantly reduced embryonic lipid accumulation within just 6 h, a level that could not be achieved by replacing one of them with L-carnitine or niacin, suggesting that simultaneously regulating cholic acid-related lipid processing and PPAR-dependent lipid metabolism may be more effective than targeting a single metabolic process. However, the current results do not confirm a direct molecular interaction between the two compounds, and further research is needed to examine lipid metabolism, fatty acid oxidation, and related signaling pathways to determine the exact metabolic basis of this combined effect.

Our findings further address whether an optimal, rather than maximal, reduction in intracellular lipid accumulation is required to maximize the cryotolerance of porcine embryos. Importantly, the present findings suggest that embryonic lipid modulation was beneficial within an appropriate metabolic range rather than through indiscriminate lipid reduction [44]. Considering the relatively fixed time window for lipid reduction treatment, we used a four-fold higher concentration of both cholic acid and bezafibrate (200 μM each) to investigate the effect of more thorough lipid clearance on porcine morula development and blastocyst cryotolerance. As a result, short-term excessively high concentrations of lipid lowering significantly reduced the conversion rate of morula to blastocyst. Since lipids are an important source of nutrition and energy in early embryos [45], we hypothesize that excessive lipid metabolism reprogramming leading to premature lipid depletion is the cause of developmental stagnation [46]. However, excessive exposure to cholic acid and bezafibrate might produce cytotoxicity, leading to mitochondrial dysfunction, apoptosis, or embryonic developmental impairment. Assessments of apoptosis and mitochondrial function, including TUNEL staining, caspase activity, and mitochondrial membrane potential analysis, were not performed in this study. Therefore, whether the developmental impairment induced by high-dose treatment was associated with apoptotic activation or mitochondrial dysfunction remains to be determined. More evidence is required to distinguish the effects of excessive lipid metabolic remodeling from cytotoxicity.

Although the pretreatment group showed a 21.43% numerical increase in pregnancy rate, the limited number of recipients precluded sufficient statistical power to establish a significant difference. Larger-scale embryo transfer studies are warranted to confirm this potential benefit. However, such studies would require substantially more donor and recipient animals and should be carefully balanced against the 3R principles of animal research. Another limitation of the present study was that follow-up after ET was limited to pregnancy and did not extend through parturition. Consequently, outcomes such as parturition, litter size, and potential genetic variation among offspring were not systematically evaluated. Future studies extending through offspring development are warranted to determine whether the effects of embryo-level pretreatment were maintained and reflected in the subsequent generation.

Last but not least, although batch production is quite mature in commercial pig farming, its application in the production and cryopreservation of local pig breed genetic resources remains relatively limited [47]. Previous studies on Meishan pigs mainly focused on individual components of the technology, such as how hormones affect early embryos [48], evaluations of vitrification using the open pulled straw method [49] or non-surgical ET [50]. In contrast, an integrated and repeatable pipeline combining donor synchronization, superovulation, artificial insemination, surgical embryo recovery, vitrification, and embryo transfer for batch production and conservation of Meishan embryos has been rarely documented. The in vivo embryo production–cryopreservation–ET process described in this study provides a practical technical reference for the conservation and utilization of local varieties.

## 5. Conclusions

The combination of bezafibrate and cholic acid reduced lipid contents in porcine embryos and improved their cryotolerance. This treatment was also associated with a numerically higher pregnancy rate after ET. This study provides a strategy for improving embryo cryopreservation by utilizing the time window between in vivo embryo production and cryopreservation.

## Figures and Tables

**Table 1:** Efficiency of batch in vivo embryo production of Meishan pigs.

Indicators of In Vivo Embryo Production	Results
Number of donors	40
Synchronization rates	95.00% (38/40)
Fertility rates	100% (38/38)
Totally embryos retrieved	859
Average number of embryos per sow	22.61
Ratio of morulae in all embryos	90.92% (781/859)

## Data Availability

Data from this study are presented in this paper and are also available from the corresponding author.

## Abbreviations

The following abbreviations are used in this manuscript:

hCG	Human chorionic gonadotropin
eCG	Equine chorionic gonadotropin
ET	Embryo transfer
FSH	Follicle-stimulating hormone
FXR	Farnesoid X receptor
GPBAR1	G protein-coupled bile acid receptor 1
NCSU23	North Carolina State University-23 medium
PPAR	Peroxisome proliferation-activating receptor
PGC-1α	Peroxisome proliferator-activated receptor gamma coactivator 1-alpha
PZM-3	Porcine zygote medium-3
TL-PVA	Tyrode’s lactate–HEPES–polyvinyl alcohol medium
